# Separation of n-Butanol from Aqueous Solutions via Pervaporation Using PDMS/ZIF-8 Mixed-Matrix Membranes of Different Particle Sizes

**DOI:** 10.3390/membranes13070632

**Published:** 2023-06-29

**Authors:** Ali Zamani, Jules Thibault, Fatma Handan Tezel

**Affiliations:** Department of Chemical and Biological Engineering, University of Ottawa, Ottawa, ON K1N 6N5, Canada; ali.zmn@hotmail.com (A.Z.); jules.thibault@uottawa.ca (J.T.)

**Keywords:** mixed-matrix membranes, biobutanol separation, ZIF-8 nanoparticles, PDMS, flux, selectivity

## Abstract

The use of mixed matrix membranes (MMMs) to facilitate the production of biofuels has attracted significant research interest in the field of renewable energy. In this study, the pervaporation separation of butanol from aqueous solutions was studied using a series of MMMs, including zeolitic imidazolate frameworks (ZIF-8)-polydimethylsiloxane (PDMS) and zinc oxide-PDMS mixed matrix membranes. Although several studies have reported that mixed matrix membranes incorporating ZIF-8 nanoparticles showed improved pervaporation performances attributed to their intrinsic microporosity and high specific surface area, an in-depth study on the role of ZIF-8 nanoparticle size in MMMs has not yet been reported. In this study, different average sizes of ZIF-8 nanoparticles (30, 65, and 80 nm) were synthesized, and the effects of particle size and particle loading content on the performance of butanol separation using MMMs were investigated. Furthermore, zinc oxide nanoparticles, as non-porous fillers with the same metalcore as ZIF-8 but with a very different geometric shape, were used to illustrate the importance of the particle geometry on the membrane performance. Results showed that small-sized ZIF-8 nanoparticles have better permeability and selectivity than medium and large-size ZIF-8 MMMs. While the permeation flux increased continuously with an increase in the loading of nanoparticles, the selectivity reached a maximum for MMM with 8 wt% smaller-size ZIF-8 nanoparticle loading. The flux and butanol selectivity increased by 350% and 6%, respectively, in comparison to those of neat PDMS membranes prepared in this study.

## 1. Introduction

Global energy demands are primarily met through non-renewable sources, such as oil, coal, and natural gas. However, the scarcity and rising prices of fossil fuels, coupled with the environmental problems facing society, stimulate the vigorous search for biofuels as viable alternatives to petroleum and other non-renewable fuel products. Biofuels have numerous advantages that can accelerate their adoption worldwide. They open the door of opportunity for renewable transportation fuels, reduction of GHG emissions and broadening of the global market for agricultural products [1,2]. Production of biofuels could also improve the economic status of local workers by providing employment opportunities. Of all liquid biofuel alternatives, biobutanol recently received increased attention as a plausible green fuel for the partial replacement of petroleum-based fuels as it possesses fuel properties that are close to those of gasoline [3]. Biobutanol has desirable fuel characteristics, such as low water solubility and vapor pressure, high energy density and cetane number [4]. It can be used directly in existing car engines without any modifications to the engines. It has high miscibility favoring its efficient blending with other fuels. As a result, biobutanol is well-suited for automotive fuel applications [5].

In 1912, Chaim Weizmann discovered a microorganism called *Clostridium acetobutylicum*, which was able to ferment starch to acetone, butanol, and ethanol [6]. The *Clostridium* bacteria is a strict anaerobe and spore-forming microbe and has been used since to produce various bio-solvents by anaerobic fermentation of numerous monosaccharides and hydrolysate of oligosaccharides [7]. This process, which is called ABE fermentation, can produce acetone, butanol, and ethanol in a typical ratio of 6:3:1, respectively. In this fermentation, 9–12 g·L^−1^ butanol is typically produced, while 40–60 g·L^−1^ glucose is consumed under batch conditions [7,8]. Various cellulosic feedstocks are currently being used for biobutanol production, such as wheat and barley straw, corn stover, cassava bagasse, switchgrass, and miscanthus, in addition to expensive edible feedstocks like glucose and corn starch [8]. Even though improvements have been achieved in this process, currently, the ABE fermentation process cannot compete on a commercial scale with petroleum-based butanol produced by the hydrolysis of haloalkanes or hydration of alkenes [9]. There are significant challenges to producing this alcohol as an economically viable biofuel. The most important challenge is the inhibition effect on the microorganism by the solvent product at concentrations in the vicinity of 1 wt% [10]. Because of the low butanol concentration caused by butanol toxicity, the economics of the process is greatly hampered when energy-intensive separation methods, such as distillation, are used for separating butanol from its dilute aqueous solution [11].

To address the low-concentration butanol challenge, it has been recommended to resort to in situ separation techniques to partially remove solvent products, especially butanol, as the most toxic product, during the fermentation process. This in situ removal allows for obtaining higher overall solvent concentrations and increases productivity [12]. Technologies such as gas stripping, liquid-liquid extraction, vacuum stripping, membrane distillation and adsorption can be used in butanol in situ separation. Another interesting technique to separate butanol from dilute aqueous mixtures is via membrane pervaporation (PV). In membrane pervaporation, a liquid mixture is in contact with a dense membrane on the feed side and some species are allowed to permeate to the other side of the membrane, where they are removed as vapor. The permeate side of the membrane is held under a vacuum, or sweep gas is used to maintain a very low pressure in order to create a concentration gradient across the membrane [13]. Polymeric membranes have been the most frequently used pervaporation membranes due to their low cost, ease of manufacture and ability to scale up.

Polydimethylsiloxane (PDMS) is recognized as a benchmark for alcohol-selective membrane material [14]. It is the most commonly used hydrophobic membrane material in PV processes due to its significant advantages of good film-forming ability, high permeability to small molecules and chemical stability. Unfortunately, the performance of polymeric membranes, such as PDMS, is limited by the well-known trade-off effect between permeability and selectivity, where an increase in the former results in a decrease in the latter and vice versa [15]. In recent years, research has focused on fabricating mixed matrix membranes (MMMs) to reduce this trade-off effect. Mixed matrix membranes are manufactured by incorporating various porous or nonporous fillers into the polymer matrix to manipulate the synergistic effect between the polymer and filler phases [16]. A wide range of porous fillers, including zeolites [17,18], metal–organic frameworks (MOFs) [19,20,21], activated carbons (ACs) [22], and carbon nanotubes (CNTs) [23], as well as nonporous fillers, such as pearl [24], silica [25] and Zinc Oxides [26] have been used in mixed matrix membranes. The permeation mechanism prevailing in mixed matrix membranes is not yet completely known. In an attempt by Cseri et al., the contribution of the MOF pore size in mixed matrix membrane was studied, and it was hypothesized that some porous fillers offer a lower resistance for some diffusing species and may provide molecular sieving depending on their pore sizes [27]. ZIF-8 is one of the most widely investigated Zeolitic Imidazolate Frameworks (ZIFs) among the metal–organic frameworks family and has extraordinary advantages in alcohol separation [28,29]. ZIF-8 possesses a sodalite (SOD) zeolite-type structure with a small pore aperture of a nominal diameter of ~3.4 Å and an effective aperture size in the range of 4.0–4.2 Å due to its flexible framework [30]. ZIF-8 could form preferential butanol channels contributed by a sub-nanometer scale hydrophobic interconnected structure that is favorable for fast butanol transport. Furthermore, ZIF-8 has additional advantages, namely its low cost, raw materials availability, simple preparation method, and tunable size and morphology [31]. Moreover, ZIF-8 shows high water stability, which is vital for water-related applications [32]. It is known that the separation performances of ZIF-8/PDMS mixed matrix membranes highly depend on the intrinsic properties of the ZIF-8 and the PDMS, the interaction between the two and the percentage of ZIF-8 loading in the mixed matrix membrane [33]. In a study by Bai et al., the effect of ZIF-8 loading up to 5 wt% in mixed matrix membranes and operating temperature between 30 °C to 60 °C was studied [34]. However, the possible effect of the ZIF-8 particle size on the permeation characteristics of the ZIF-8-filled polymeric membranes has not yet been investigated. In this study, higher loadings of ZIF-8/PDMS were prepared to seek optimum performance for the separation of biobutanol using mixed matrix membranes. In addition, different particle sizes of ZIF-8 were synthesized and incorporated into the matrix of the PDMS membranes to study the effects of the particle size on the performance of PDMS MMMs for the separation of butanol from aqueous binary solutions.

## 2. Materials and Methods

### 2.1. Materials

Polydimethylsiloxane (PDMS) and cross-linking agent kit (RTV615 001 KIT) were obtained from Momentive Co. (Hebron, OH, USA). Polyacrylonitrile (PAN) membranes, used as a support for PDMS in this study, were purchased from Synder Filtration (Vacaville, CA, USA) with 30,000 Da molecular weight cut-off and a thickness of 0.15 mm (Polyester + PAN). Zinc Nitrate Hexahydrate (Zn(NO_3_)_2_·6H_2_O), 2-Methylimidazole (Hmim, 99%) and Sodium Hydroxide (NaOH) were purchased from Sigma Aldrich (Ottawa, ON, Canada). Methanol (99.9% purity), Butanol (99% purity) and Tetrahydrofuran (THF) were obtained from Fisher Scientific Inc. (Ottawa, ON, Canada). All reagents were used as received without further purification, and deionized distilled water was used to prepare all aqueous solutions.

### 2.2. Synthesis of Nanoparticles

#### 2.2.1. Synthesis of ZIF-8 Nanoparticles

ZIF-8 was synthesized based on a similar synthesis protocol described by Demir et al. [35], using Zinc Nitrate Hexahydrate (Zn(NO_3_)_2_·6H_2_O), 2-Methyl Imidazole (Hmim), and methanol. In a typical synthesis, 2.4 g of Zinc Nitrate Hexahydrate and 5.3 g of Hmim were dissolved in separate beakers containing 90.4 g of methanol by stirring at room temperature for 15 min at 300 rpm. These two beakers were then combined gradually into one to initiate the synthesis of the ZIF-8 particles, forming a milky white solution. ZIF-8 synthesis was then completed by stirring for 1 h at 300 rpm at room temperature. The particles were then separated from the synthesis solution by centrifugation at 6000 rpm for 5 min. The resulting supernatant liquid, referred to as the mother liquor, was put aside and recycled to produce more ZIF-8 particles with the addition of sodium hydroxide. The separated particles were then washed with deionized distilled water and re-separated by centrifugation at 6000 rpm for 20 min. The washing-centrifugation process was performed three times. The particles were then placed in an oven and dried at 80 °C for 12 h. Low-temperature calcination, which is sometimes referred to as annealing, was then conducted by placing the particles in a preheated oven at 180 °C for 12 h. At the end of the 12 h, the particles were taken out of the oven and cooled down to room temperature.

For the ZIF-8 particles fabricated in this study, ZIF-8-1 particles were fabricated using the above procedure. ZIF-8-2 particles were fabricated using the mother liquor from the fabrication of ZIF-8-1 particles in combination with additional solid NaOH enough to increase the pH from approximately 7.0 to 9.0 as the synthesis solution. Furthermore, ZIF-8-3 particles were fabricated using the mother liquor from the fabrication of particles ZIF-8-2 with added Zinc Nitrate Hexahydrate as the synthesis solution. The Zinc Nitrate Hexahydrate added in this step was equal to the initial amount of 2.4 g. The synthesis solutions for ZIF-8-2 and ZIF-8-3 particles were then aged at room temperature by stirring the solution for one hour at 300 rpm. The particles were then separated, washed, dried, and calcined the same way as for ZIF-8-1 particles in this study. It is also worth mentioning that there are more sustainable ways to synthesize ZIF-8, such as the procedures suggested by Hardian et al. that follow United Nations’ Sustainable Development Goals [36].

#### 2.2.2. Synthesis of Zinc Oxide (ZnO) Particles

ZnO particles were prepared using the method outlined previously by Wu et al. [37] with some modifications. In this method, the alkali solution of zinc was prepared by dissolving 2.5 g of Zinc Nitrate Hexahydrate and 3.5 g NaOH in two separate 80 mL deionized water at room temperature. Then, the NaOH solution was heated to 30 °C, and the Zinc Nitrate solution was added dropwise under constant stirring. After stirring for 2 h at 300 rpm and 30 °C, the white precipitate that settled at the bottom of the beaker was collected by centrifugation at 6000 rpm for 5 min. The ZnO particles were then washed with methanol and re-separated by centrifugation two more times before being dried at 80 °C for 12 h.

### 2.3. Fabrication of Membranes

#### 2.3.1. Neat Membrane

Neat PDMS membranes were prepared using the method outlined by Azimi et al. [13]. PAN membranes, which are known to have high porosity, were used as a backing material to deposit a thin PDMS layer. After rinsing the PAN thoroughly with water, it was taped on a piece of clean glass to hold it in place. The PDMS solution for the active layer was prepared by mixing 5 g of the base PDMS from the silicone kit in 20 g of THF. The solution was mixed using a stirrer (RZR 2102, Heidolph Electronic, Wood Dale, IL, USA) for 30 min, and then 0.5 g of the crosslinking agent was added to this mixture and stirred for an additional 45 min. The PDMS solution was then sprayed onto the PAN membrane using an air pen brush (Paasche VL-SET Double Action Siphon Feed Airbrush, Kenosha, WI, USA) in two successive layers. The main solution was first sprayed as uniformly as possible in one direction onto the PAN support, and, following a 10 min period under vacuum condition, the membrane was turned 90° and a second layer was sprayed akin to the first layer. The glass plate with the membrane was then placed in a vacuum oven. The vacuum oven was maintained at an absolute pressure of 0.2 bar for 30 min at room temperature, and then the oven was heated up to 90 °C for 3 h, including the pre-heating period, while maintaining the same vacuum pressure. Following this curing procedure, the membrane was taken out of the oven and cooled to room temperature.

#### 2.3.2. ZIF-8 and ZnO Nanoparticles Filled PDMS Mixed Matrix Membranes

To fabricate the mixed matrix membranes, a procedure similar to the one mentioned in Section 2.3.1 for the neat PDMS membrane was followed. However, a certain weight percent of ZIF-8 nanoparticles was added to the main solution for the preparation of the membrane. The nanoparticle percentages were evaluated using Equation (1).

(1) $$\mathrm{ZIF}-8\;\left(\mathrm{wt}\%\right)=\frac{W_{ZIF-8}}{W_{ZIF-8}+W_{PDMS}}\times 100$$

where *W_ZIF-_*_8_ and *W_PDMS_* are the weights of the nanoparticle and the base PDMS from the silicon kit, respectively, in the membrane casting solution.

The nanoparticles were first thoroughly mixed within 20 g of THF using a sonicator (QSONICA, Part No. Q700, Newtown, CT, USA) at 0 °C temperature for 1 h. Then, 5 g of PDMS from the silicon kit was added to the mixture and mixed for 30 min at 300 rpm at room temperature. Next, 0.5 g of the crosslinking agent was added and stirred for 45 min at 300 rpm at room temperature. The same procedure described in Section 2.3.1 was then used to apply the two successive layers of the ZIF-8/PDMS solution, including the subsequent curing of the membrane. Noteworthy, the spray nozzle was large enough to spray the solution without any clogging to ensure that the ZIF-8/PDMS solutions were sprayed uniformly. The same procedure was used to fabricate ZIF-8/PDMS and ZnO/PDMS membranes with different particle sizes.

### 2.4. Characterization of Nanoparticles and Membranes

Images of the membranes were taken using a scanning electron microscope (SEM, JSM-7500F, Peabody, MA, USA). Each sample was freeze-fractured after immersion in liquid nitrogen and was then taped on support using carbon tape to fix the sample. The sample was gold sputtered before SEM observations were made. Powder X-ray diffraction (XRD) patterns of ZIF-8 particles were acquired using a Max Rigaku X-ray diffractometer with a copper anode, and a graphite monochromator to select Cu-Kα radiation (λ = 1.540 Å), taking data from 2θ=0° to 80° at a scan rate of 1°/s. Transmission electron microscopy (TEM) was performed on an FEI Tecnai G2 Spirit Twin and operated at 120 kV. The samples were dispersed in THF and loaded on 50 nm 300-mesh carbon-coated copper grids.

A digital micrometer caliper (0-1”, Mitutoyo, Aurora, IL, USA) was used to measure the membrane thickness. Measurements were made at five different spots, and the average was reported. The hydrophobicity/organophilicity of the membrane surface was characterized by the static and dynamic contact angle measurements. The static contact angle (SCA) was measured using the video optima surface analysis system (Optima AST Product Inc., Billerica, MA, USA) by placing a 3 μL droplet of the solution on the membrane surface.

The machine is able to capture static and dynamic pictures of the droplet and calculates the surface contact angle by determining the tangent lines. For each membrane, the static contact angle was measured at five different locations, and the values were averaged. The static contact angle was measured for pure water and for a 5 g·L^−1^ butanol aqueous solution. The dynamic contact angle of pure butanol was measured by recording 10 frames for 1 min considering the rapid change of butanol droplet contact angle at the surface due to butanol evaporation and impregnation.

#### PV Experiments

**Figure 1:**
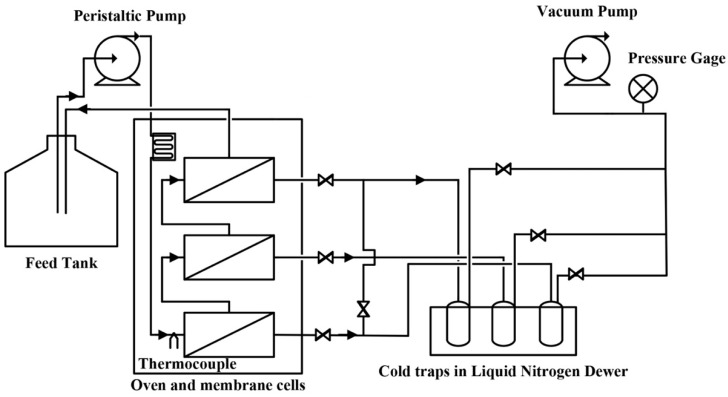
Schematic diagram of the pervaporation experimental setup. Reprinted with permission from Ref. [22]. 2017, *J. Chem. Technol. Biotechnol*.

Permeation experiments were carried out using the pervaporation experimental setup presented in Figure 1 [22]. The setup consists of three membrane modules placed in series where the retentate from each membrane is directed to the next one. The three-module membrane system was placed in a temperature-controlled oven accompanied by a long stainless-steel coil to ensure the feed stream reached the desired temperature prior to entering the first membrane module. Furthermore, a thermocouple was used to measure the feed temperature halfway between the coil and the first membrane to confirm that the temperature reached its desired value. The vapor phase streams exiting the permeate side of each membrane module were collected in individual cold traps. Cold traps were immersed into liquid nitrogen Dewar accompanied by an automatic time-fill controller to maintain a pre-set liquid nitrogen level (Gordinier Electronics Inc, model 359 liquid time fill, Roseville, MI, USA). The permeate side of the three membrane modules and the three cold traps were maintained at very low pressure (1 Torr) using a vacuum pump (Scroll Pump, 78603-11, Cole-Parmer, Montreal, QC, Canada). A digital pressure gauge was used after the cold traps to monitor the vacuum pressure. At the end of each experiment, the permeation rate was determined gravimetrically by weighing the permeate sample collected over a given period of time. Both the feed and permeate compositions were analyzed by a liquid density meter (DMA 4500 M, Anton Paar, Saint-Laurent, QC, Canada). In some cases, the content of butanol in the permeate exceeded its solubility limit at room temperature, and the permeate sample formed two phases. Under these circumstances, the permeate sample was diluted with deionized water when determining the overall permeate composition. Initially, the butanol concentration in the feed was fixed at 5 g·L^−1^. During a pervaporation run, the quantity of permeate removed by the membrane was kept below 1% of the initial feed load to maintain an essentially constant feed composition. Since the reference feed temperature of ABE fermentation broth is usually between 37–40 °C, the feed temperature was considered constant at 39 °C. All the experimental data reported were obtained at steady-state pervaporation.

The permeation flux, selectivity and permeability were used to characterize the membrane performance. The flux (*J*) is the permeate flow rate per unit membrane surface area, which is normally determined for each species from the total permeation flux and permeate mass fraction of each component. The membrane selectivity can be characterized by the separation factor (*α*), which is a metric that assesses the separation ability of the membrane considering two substances to be separated. The permeability (*P*) allows the comparison of the membranes’ performance with different properties.

These performance parameters for individual species *i* are defined in Equations (2)–(4).

(2) Ji=miA·t

(3) αi=yi1−yixi1−xi

(4) $$P_{i}=\frac{J_{i}\cdot \sigma }{C_{i-feed}-C_{i-permeate}}$$

where *m_i_* is the mass of component *i* in the permeate stream (kg), *A* is the effective surface area of the membrane (m^2^), *t* is the time of permeation (s), *y_i_* and *x_i_* are the mass fraction of component *i* in the permeate and feed streams, respectively. *P_i_* is the permeability (m^2^ s^−1^), and *σ* is the effective thickness of the membrane (m). $C_{i-feed}$ and $C_{i-permeate}$ are the concentrations of component *i* in the feed and permeate side, respectively (kg·m^−3^). By assuming a high vacuum on the permeate side, it can be assumed that $C_{i-permeate}$ is equal to zero.

## 3. Results and Discussion

### 3.1. Characterization of ZIF-8 and ZnO Nanoparticles

**Figure 2:**
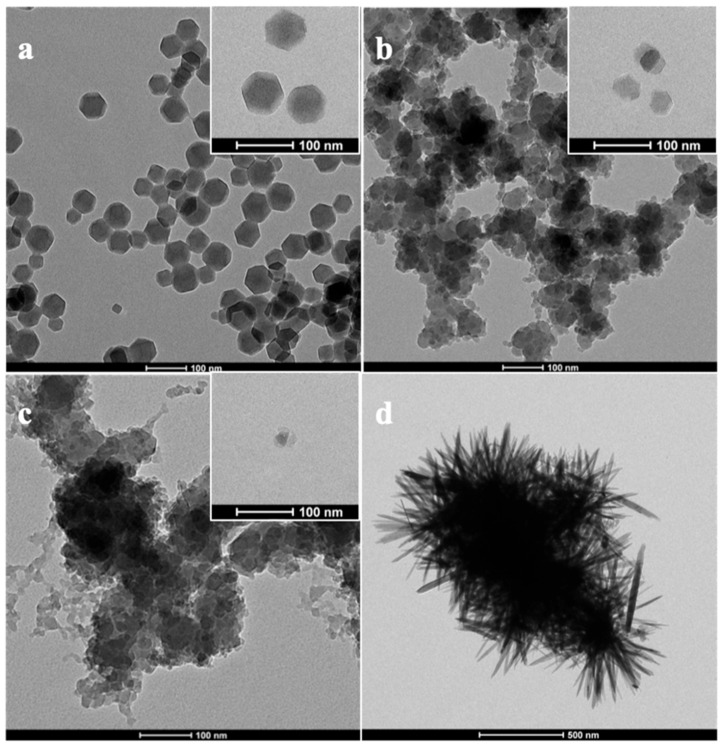
TEM images for (**a**) ZIF-8-1 nanoparticles, (**b**) ZIF-8-2 nanoparticles, (**c**) ZIF-8-3 nanoparticles and (**d**) ZnO particles.

ZnO and three different sizes of ZIF-8 (i.e., ZIF-8-1, ZIF-8-2, ZIF-8-3) were synthesized and characterized before using them as fillers in PDMS-based MMMs. Representative TEM images of the different samples are presented in Figure 2, where the different particle sizes and morphologies are compared. While the smallest ZIF-8 particles (ZIF-8-3) are almost spherical, the largest particles (ZIF-8-1) have well-defined edges with the typical rhombic dodecahedron shape of ZIF-8 that has been suggested by Demir et al. [35]. In addition, the rod-like shape of ZnO is consistent with the geometry that was reported in the literature [37]. A statistical size evaluation using more than 100 nanoparticles of ZIF-8 indicates that the average size of ZIF-8-1, ZIF-8-2 and ZIF-8-3 nanoparticles were approximately 80 ± 20, 65 ± 8 and 30 ± 15 nm, respectively, as listed in Table 1. The relatively small particle sizes of all three ZIF-8 are considered an advantage for the successful fabrication of defect-free mixed matrix membranes.

**Figure 3:**
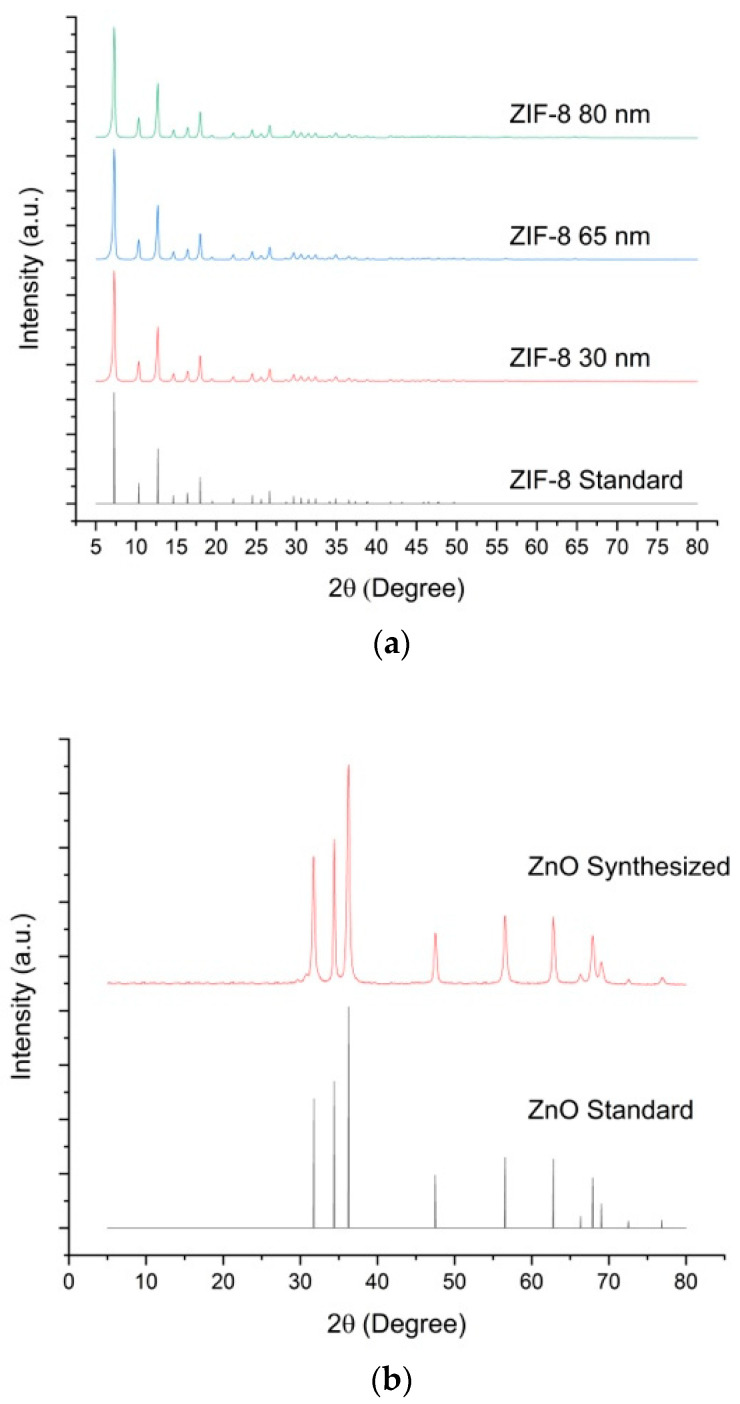
(**a**) XRD patterns of ZIF-8, which were synthesized in different particle sizes. The ZIF-8 standard data represent the simulation pattern obtained from Cambridge Crystallographic Data Center. (**b**) XRD patterns of synthesized ZnO particles. The ZnO standard data represent the simulation pattern obtained from Cambridge Crystallographic Data Center.

The XRD patterns of ZIF-8-1, ZIF-8-2 and ZIF-8-3 MOFs with sizes between 30 and 80 nm are represented and compared to the ZIF-8 standard in Figure 3a. All samples indicate a pattern with identical shapes and peaks at the same positions, acknowledging that they constitute the same kind of MOF. Moreover, the height and width of the different peaks are equivalent for all samples, disregarding their size, which also indicates high crystallinity. Furthermore, the XRD pattern for ZnO is provided in Figure 3b to ensure identical crystallinity with the standard.

### 3.2. Characterization of ZIF-8/PDMS MMMs

#### 3.2.1. Scanning Electron Microscopy (SEM) Results

**Figure 4:**
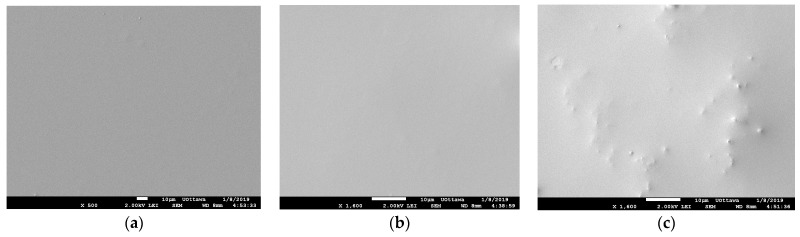
SEM images of the surface of the membrane: (**a**) Neat PDMS, (**b**) 8 wt% ZIF-8-1/PDMS and (**c**) 8 wt% ZnO/PDMS.

SEM was used to visualize the surface and cross-sectional structure of the membranes prepared. As shown in Figure 4, the pure PDMS and 8 wt% ZIF-8-1/PDMS membranes have smooth surfaces compared to 8% ZnO/PDMS membranes. ZIF-8/PDMS smoothness confirms a homogeneous and defect-free MMM surface, as well as good interface compatibility between ZIF-8 and the hydrophobic PDMS phase. On the other hand, the complex rod-like shape of ZnO may contribute to the rough surface of ZnO/PDMS membranes, which led to a larger surface area. The SEM surface image analysis was also performed for all the other MMMs including different ZIF-8 particle sizes and since their surface images were similar and as smooth as pure PDMS, those SEM surface pictures are not included in Figure 4.

**Figure 5:**
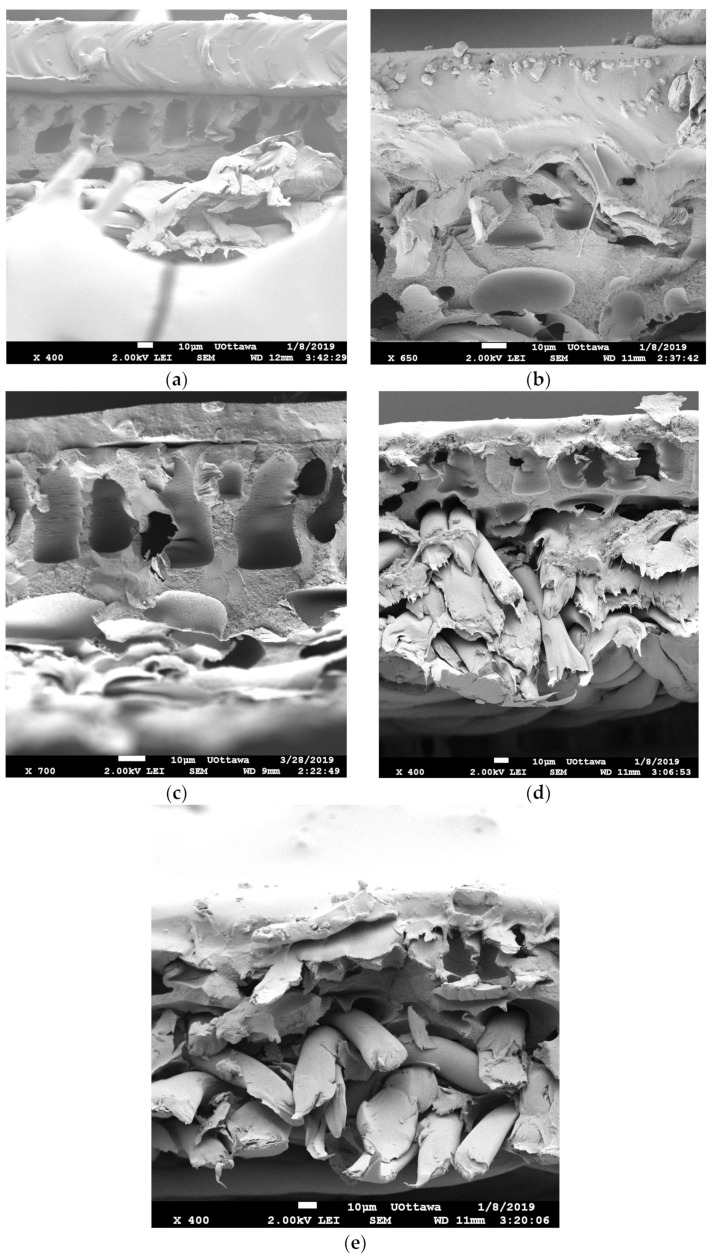
SEM cross-section images of (**a**) neat PDMS, (**b**) 8 wt% ZIF-8-1/PDMS, (**c**) 8 wt% ZIF-8-2/PDMS, (**d**) 8 wt% ZIF-8-3/PDMS and (**e**) ZnO/PDMS.

Figure 5a presents the SEM image of the cross-section of the pure PDMS membrane, which consists of three layers: (1) Dense PDMS layer at the top corresponding to the active layer, (2) PAN porous support layer, (3) Polyester layer. Layers 2 and 3 are identical for all MMMs manufactured in this study and referred to as PAN support. It is worth noting that the first (active) layer is the crucial part of the membrane, while other layers provide structural support. Figure 5b shows that a certain level of agglomeration prevails on the cross-sectional area of the ZIF-8-1/PDMS MMM’s active layer, which may be attributed to the bigger particle sizes and non-uniform distribution of ZIF-8. In comparison, fewer particle agglomerations were observed in cross-sectional pictures of ZIF-8-2, ZIF-8-3, and ZnO/PDMS MMMs’ active layers. Overall, good compatibility between ZIF-8 particles and PDMS polymer chains, as well as a homogeneous distribution of smaller particle sizes in the PDMS layer, were obtained. However, as previously shown in Figure 2d, the complex geometry of ZnO, especially when agglomeration occurs, makes it difficult for the viscous PDMS solution to penetrate and cover the surface area of all the nanoparticles. This poor PDMS solution penetration leads to a higher permeability but at the expense of lower selectivity as a result of these voids and defects.

#### 3.2.2. Surface Hydrophobicity

**Figure 6:**
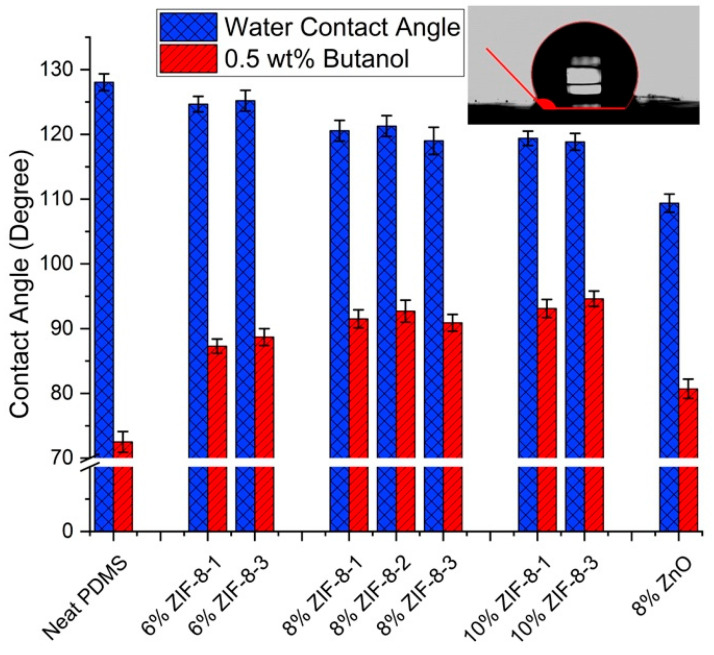
Surface static contact angle of PDMS composite membranes for pure water and 0.5 wt% butanol solution.

The static contact angle measurements were performed to investigate the hydrophobic and organophilic properties of the membranes prepared. Figure 6 presents the static contact angles for pure water and 0.5 wt% butanol aqueous solution for the neat PDMS membrane, ZIF-8/PDMS MMMs and ZnO/PDMS MMMs with different particle loadings and particle sizes. The pure PDMS membrane had a static water contact angle of 128° that shows its favorable intrinsic hydrophobicity. There were no significant differences in the water contact angle between different particle sizes of ZIF-8/PDMS MMMs, with slightly lower values than those for the neat PDMS membrane. The water contact angle continued to decrease with increasing ZIF-8 loading, indicating the diminished hydrophobicity of the PDMS membrane after incorporating ZIF-8 particles. In the case of the ZnO/PDMS membrane, the agglomeration of small-scale nanorods caused by particles’ geometry can affect the surface properties and eventually decrease the water contact angle. As far as the contact angle for 0.5 wt% butanol is concerned, as can be seen from Figure 6, it generally increases with more addition of ZIF-8 and the ZnO particles in the MMM.

**Figure 7:**
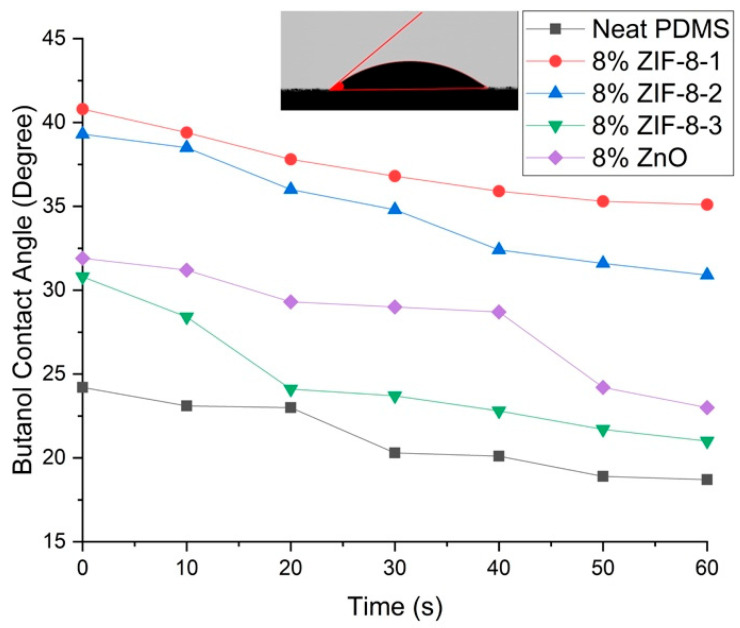
The dynamic contact angle of PDMS composite membranes for pure butanol.

Furthermore, the dynamic contact angle was used to measure the pure butanol contact angle for MMMs with different particle sizes for 1 min. As Figure 7 denotes, the neat PDMS membrane exhibited the lowest dynamic contact angle as expected, and ZIF-8-3/PDMS showed better organophilic behavior than bigger particle sizes of ZIF-8, indicating a better affinity towards butanol.

#### 3.2.3. Effect of ZIF-8 Nanoparticle Loading on the Membrane Performance

**Figure 8:**
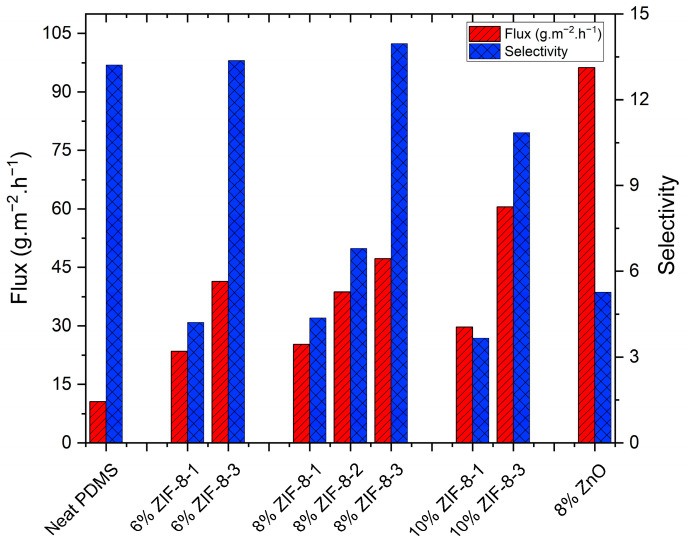
Pervaporation separation performance of 0.5 wt% butanol solution for the pure PDMS, ZIF-8/PDMS and ZnO/PDMS membranes at 39 °C.

The effect of the particle loading on the PV performance of the ZIF-8/PDMS MMMs using 0.5 wt% butanol aqueous solution at 39 °C was investigated. For each experiment, three membrane samples were used, and the averaged values of the flux, selectivity and thickness are reported in this section. Very often, the flux and the selectivity are used to assess the membrane pervaporation performance. Results for the total flux and the selectivity for the various MMMs used in this investigation are presented in Figure 8. They show that by increasing the ZIF-8-1 loading from 0 to 10 wt%, the total flux increased by 181%, while the selectivity decreased by 72% compared to the neat PDMS membranes. By incorporating porous ZIF-8 into the PDMS polymer chains, the free volume of MMMs increases, accompanied by a decrease in the mass transport resistance, thereby leading to the enhancement of the total flux. Unfortunately, the trade-off between flux and selectivity could not be broken for this particle size.

**Figure 9:**
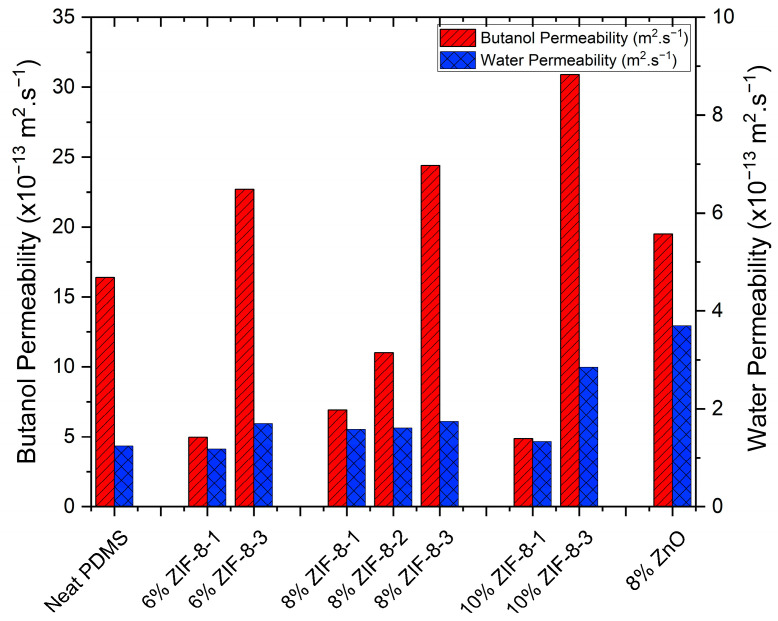
Water and butanol permeability for 0.5 wt% butanol solution for the pure PDMS, ZIF-8/PDMS and ZnO/PDMS membranes at 39 °C.

It is important to note that the flux and the selectivity are not only functions of the inherent properties of the membranes but also depend on the operating conditions, such as the feed concentration and temperature, as well as the membrane thickness. Therefore, a better way of reporting the pervaporation data is by normalizing the flux of each component by the feed concentration and the membrane thickness, which in fact, defines the membrane permeability *P_i_* as shown in Equation (4). These permeability values are shown in Figure 9. According to this figure, there was no significant increase in water permeability by adding ZIF-8-1 (larger) particles at 6% loading, even though the butanol permeability decreased compared to the neat PDMS membrane. This observation may suggest that the interfacial voids are less likely to affect the membrane performances. It is hypothesized that the PDMS chain mobility may be reduced due to the denser chain packing in the vicinity of the dispersed ZIF-8-1 particles. This phenomenon, known as the chain rigidification effect, was investigated for ZIF-8/PDMS membranes by Fang et al. [38]. It is also possible that PDMS chain segments adhere to the surrounding pores of ZIF-8 particles and cause pore blockage, which could profoundly impact the permeation of butanol by increasing the membrane diffusion resistance, resulting in low butanol permeability and selectivity.

Although there was no overall improvement by embedding ZIF-8-1 (larger) particles compared to the neat PDMS membranes at 6 % loading, 8 wt% ZIF-8-1/PDMS membranes showed higher butanol permeability (Figure 9) and selectivity (Figure 8) than membranes with 6 and 10 wt% loadings. Furthermore, zinc oxide nanoparticles, as non-porous fillers with the same metalcore as ZIF-8 but with a very different geometrical shape, were used to illustrate the importance of the particle geometry on the membrane performance. According to Figure 8, 8 wt% ZnO/PDMS membranes showed an increase of 811% in the total flux, but at the expense of a 60% decrease in the selectivity in comparison to the pure PDMS membranes. Contrary to the ZIF-8-1 (larger) particles, using non-porous ZnO in the PDMS matrix potentially eliminates the pore-blocking effect but increases the possibility of forming interfacial voids between ZnO and PDMS chains due to its complex shape. These voids favor Knudsen diffusion and lead to increased permeance, which supports the increase in the total flux and total permeability observed in Figure 8 and Figure 9, respectively. On the other hand, these Knudsen dominant voids work as non-selective pathways that benefit the water molecules, with smaller dynamic diameters than butanol, to diffuse faster and reduce the selectivity dramatically.

#### 3.2.4. Effect of the ZIF-8 Particle Size on the Membrane Performance

As previous results suggested, the 8 wt% ZIF-8-1/PDMS membranes presented a better pervaporation performance than MMMs with 6 and 10 wt% loading of ZIF-8-1 nanoparticles. In order to gain a deeper insight with respect to the influence of the filler size on the membrane performance, 8 wt% ZIF-8/PDMS MMMs were fabricated using the smaller particle sizes as ZIF-8-2 and ZIF-8-3 and tested under identical experimental conditions (initial butanol concentration of 5 g·L^−1^, at 39 °C). Results presented in Figure 8 exhibited a notable difference in MMM permeation performance. Indeed, by decreasing the particle size from 80 nm (for ZIF-8-1) to 30 nm (for ZIF-8-3), the total flux and selectivity increased 88% and 220%, respectively. Increasing both the flux and the selectivity is finally overcoming the trade-off effect associated with the majority of PDMS MMMs with enhanced separation performances. Generally, smaller particles provide a higher surface area-to-mass ratio. Therefore, a higher surface area gives a larger number of active sorption sites and provides an alternative pathway for mass transport through the inner pores of the adsorbent. These pathways act as selective channels in favor of butanol molecules and increase butanol permeability and, as a result, enhance membrane pervaporation performance.

Based on the results of Figure 9, the permeability of butanol for the MMM increased by 59% for the intermediate-size ZIF-8-2 nanoparticles and by 252% for the smaller-size ZIF-8-3 particles compared to the larger particles at 8 wt% loading for ZIF-8-1. Even though butanol permeability significantly increased when the particle size was decreased, the water permeability only increased by 1.8% and 10.2% for MMMs with ZIF-8-2 and ZIF-8-3 nanoparticles compared to MMMs with ZIF-8-1 particles, respectively.

The surface area is not the only factor affecting the membrane pervaporation performance. ZIF-8-1 nanoparticles are supposed to provide similar pathways as ZIF-8-2 and ZIF-8-3. However, it is hypothesized that the ZIF-8-1 nanoparticles may suffer from polymer rigidification and pore blockage. These polymer-particle interactions seem to decrease when smaller particle sizes are used. A study by Yin et al. [39] on ZIF-71 particles with the same Imidazole ligand as ZIF-8 reported that despite the hydrophobicity of ZIF materials, the -N-H group in Imidazole ligand still has a weak hydrophilic character. Therefore, the smaller particles, which have a higher surface area to volume ratio than the larger particles, were thermodynamically driven to agglomerate due to the nature of this hydrophilic surface. Even though particles are evenly dispersed in the polymer matrix, and no obvious particle agglomeration was observed, it is highly possible that nano-scale agglomeration occurred. These agglomerated particles have a lower interfacial connection with PDMS chains and tend to create voids instead of a pore-blocking effect. In this case, ZIF-8/PDMS membranes can actually benefit from voids to overcome pore-blocking effects, which can explain the impact of smaller particles on membrane pervaporation performance enhancement. However, if the voids increase significantly by increasing the particle loadings, as in 10 wt% ZIF-8-3 membranes, the permeability of both water and butanol would increase at the cost of sacrificing the selectivity. Furthermore, both 6 wt% ZIF-8 and 10 wt% ZIF-8/PDMS membranes showed the same behavior as 8 wt% ZIF-8/PDMS membranes by changing the particle size. The flux increased by adding up to 10 wt% ZIF-8-3 particles to the polymer matrix, but the selectivity only increased up to 8 wt% ZIF-8-3 particle loading, and a sudden decrease is observed at 10 wt%.

The best membrane pervaporation separation performance achieved in this study was associated with the 8 wt% ZIF-8-3/PDMS mixed matrix membranes where the flux and the selectivity were increased up to 350% and 6%, respectively, compared to neat PDMS membranes, as can be seen in Figure 8.

## 4. Conclusions

The ultimate goal of this study was to investigate the effect of the filler size on MMM performance for the separation of butanol from aqueous binary solutions, which has been achieved by successfully synthesizing different sizes of ZIF-8 nanoparticles (30, 65, and 80 nm) and conducting pervaporation experiments. While butanol permeability, flux and selectivity increased with a decrease in ZIF-8 particle size, water permeability remained constant. Furthermore, ZnO/PDMS membranes were investigated to emphasize the importance of interface compatibility between the filler and the polymer chains.

The impact of the ZIF-8 particle loading on the pervaporation separation of butanol from aqueous solution has been studied for three different ZIF-8 sizes. An increase in the ZIF-8 loading content in the MMM with PDMS up to 10 wt% increased the total flux. The maximum selectivity was observed at 8 wt% loading of the small-sized ZIF-8 nanoparticles in the PDMS. The total permeation flux and the butanol selectivity were increased up to 350% and 6%, respectively, for the 8 wt% small-size ZIF-8/PDMS mixed matrix membranes compared to the neat PDMS membrane.

## Figures and Tables

**Table 1:** Different particle sizes of ZIF-8 and ZnO were synthesized in this study.

Particle Name	Particle Size (nm)
ZIF-8-1	80 ± 20
ZIF-8-2	65 ± 08
ZIF-8-3	30 ± 15
ZnO	500 ± 100

## Data Availability

The data presented in this study are available on request from the corresponding author.

## Abbreviations

ABE	Acetone: Butanol, Ethanol
AC	Activated Carbon
CNT	Carbon nanotube
MMM	Mixed Matrix Membrane
MOF	Metal organic framework
PAN	Polyacrylonitrile
PV	Pervaporation
PDMS	Polydimethylsiloxane
SCA	Static contact angle
SEM	Scanning Electron Microscope
SOD	Sodalite
TEM	Transmission Electron Microscopy
THF	Tetrahydrofuran
XRD	X-ray Powder Diffraction
ZIF	Zeolitic Imidazolate Framework

## Nomenclatures

*A*	Surface area of the membrane (m^2^)
*C_i−feed_*	Concentration of species i in the feed stream (kg·m^−3^)
*C_i−permeate_*	Concentration of species i in the permeate stream (kg·m^−3^)
*J*	Flux (kg·m^−2^·s^−1^)
*m_i_*	Mass of species i in the permeate stream (kg)
*P_i_*	Membrane permeability of species i (m^2^·s^−1^)
*t*	Time of permeation (h)
*W_PDMS_*	Weight of the PDMS polymer (g)
*W_ZIF-8_*	Weight of the ZIF-8 nanoparticles (g)
*x_i_*	Mass fraction of species i in the feed stream (g_i_·g^−1^_solution_)
*y_i_*	Mass fraction of species i in the permeate stream (g_i_·g^−1^_solution_)
*σ*	Membrane effective thickness (m)
*α_i_*	Selectivity of species i (-)
